# Mortality estimates among adult patients with severe acute respiratory infections from two sentinel hospitals in southern Arizona, United States, 2010–2014

**DOI:** 10.1186/s12879-018-2984-1

**Published:** 2018-02-12

**Authors:** Steve R. Barnes, Zimy Wansaula, Kristen Herrick, Eyal Oren, Kacey Ernst, Sonja J. Olsen, Mariana G. Casal

**Affiliations:** 1 Arizona Department of Health Services, Border Infectious Disease Surveillance Program, 400 West Congress, Suite 116, Tucson, AZ 85701 USA; 2 Mel and Enid Zuckerman College of Public Health, University of Arizona, 1295 N Martin Ave, Tucson, AZ 85724 USA; 3 Arizona Department of Health Services, Office of Infectious Disease Services, 150 N 18th Ave Phoenix, Phoenix, AZ 85007 USA; 4 Influenza Division, Centers for Disease Control and Prevention, 1600 Clifton Road, Atlanta, GA 30329-4027 USA

**Keywords:** Arizona, Death certificates, Fatal outcome, Influenza, Respiratory tract diseases, Surveillance

## Abstract

**Background:**

From October 2010 through February 2016, Arizona conducted surveillance for severe acute respiratory infections (SARI) among adults hospitalized in the Arizona-Mexico border region. There are few accurate mortality estimates in SARI patients, particularly in adults ≥ 65 years old.

The purpose of this study was to generate mortality estimates among SARI patients that include deaths occurring shortly after hospital discharge and identify risk factors for mortality.

**Methods:**

Patients admitted to two sentinel hospitals between 2010 and 2014 who met the SARI case definition were enrolled. Demographic data were used to link SARI patients to Arizona death certificates. Mortality within 30 days after the date of admission was calculated and risk factors were identified using logistic regression models.

**Results:**

Among 258 SARI patients, 47% were females, 51% were white, non-Hispanic and 39% were Hispanic. The median age was 63 years (range, 19 to 97 years) and 80% had one or more pre-existing health condition; 9% died in hospital. Mortality increased to 12% (30/258, 30% increase) when electronic vital records and a 30-day post-hospitalization time frame were used. Being age ≥ 65 years (OR = 4.0; 95% CI: 1.6–9.9) and having an intensive care unit admission (OR = 7.4; 95% CI: 3.0–17.9) were independently associated with mortality.

**Conclusion:**

The use of electronic vital records increased SARI-associated mortality estimates by 30%. These findings may help guide prevention and treatment measures, particularly in high-risk persons in this highly fluid border population.

## Background

Pneumonia and influenza remain leading causes of morbidity and mortality in the United States [1]. Adults aged 65 years and older, children under five years, pregnant women and those with chronic medical conditions such as asthma or heart disease are at high risk for complications that might lead to hospitalization and death [2–5]. Each year, influenza causes an estimated 140,000–710,000 hospitalizations and 12,000–56,000 deaths in the United States alone [6]. In 2015, the pneumonia and influenza mortality rate was estimated at 18/100,000 among U.S. residents of all ages and 102/100,000 in those aged ≥ 65 years [7]. In 2015, among Arizona residents, the pneumonia and influenza mortality rate was estimated at 11/100,000 and 58/100,000 for all age groups and adults aged ≥ 65 years, respectively [7, 8].

The 2009 H1N1 influenza pandemic highlighted the need to better understand severe influenza in persons admitted to a hospital. In response, the World Health Organization (WHO) recommended enhanced influenza surveillance guidelines and implementation of surveillance for severe acute respiratory infections (SARI) in member countries [9, 10]. The Arizona Department of Health Services (ADHS) has conducted statewide influenza surveillance since 1997. Surveillance activities include monitoring influenza-like illness (ILI) among ambulatory patients, tracking laboratory-confirmed cases, monitoring ILI in sentinel schools, and testing and subtyping influenza viruses in specimens submitted to the Arizona State Public Health Laboratory. In 2010, in response to the WHO guidance, ADHS began conducting sentinel surveillance for SARI as part of the Centers for Disease Control and Prevention (CDC) Border Infectious Disease Surveillance program (BIDS) with the aim of describing the patterns of severe disease in a highly fluid border region. SARI surveillance was established in Pima County, Arizona along the U.S.-Mexico Border, and aimed to complement existing statewide influenza surveillance activities. Previous estimates of mortality among SARI patients in this population have been limited to in-hospital mortality, thus, deaths that occurred after hospital discharge were not captured [11]. The main objective of this analysis was to quantify mortality among adult patients hospitalized with SARI by capturing deaths occurring both in hospital and shortly after hospital discharge, and to identify potential risk factors for mortality.

## Methods

This analysis was exempt from ethical approval because ADHS’ Human Subjects Review Board determined that SARI surveillance was part of public health practice.

### Study sites

Between October 2010 and February 2016, SARI surveillance was conducted at three sentinel sites in Pima County that routinely receive acutely ill patients and transferred patients from smaller hospitals in the border region. Selection of study sites has been described in detail elsewhere [11]. In brief, the ADHS, BIDS program identified hospitals in the four Arizona counties (Cochise, Pima, Santa Cruz, and Yuma) that border Mexico and receive mobile, foreign born and migrant populations. There were 11 acute care hospitals within this area; surveillance was initiated at five hospitals in 2010. Two of the participating hospitals had fewer than five participants in the first season, leaving three in the surveillance system. One of the three sentinel surveillance sites did not have data from electronic records accessible; therefore, only two sites were included in this mortality analysis. This analysis was limited to SARI patients who presented to the inpatient ward of St. Mary’s Hospital or St. Joseph’s Hospital in Pima County, Arizona. These two large acute care sites transfer pediatric cases to other regional hospitals; therefore, only adults ≥18 years were included in this analysis. For this analysis, we defined each influenza season as beginning in week 40 and ending in week 39 of the consecutive year.

### Case ascertainment and data collection methods

The details of the SARI surveillance methods have been described in detail elsewhere [11]. In brief, clinical teams at each surveillance site were trained on the SARI case definition (Additional file 1: Table S1) and surveillance procedures. Patients who presented at the emergency ward who were identified by the clinical team as meeting the SARI case definition were asked for verbal consent to participate. Data for enrolled patients were collected using a standardized case report form. Data collected included age, sex, race/ethnicity, residence, symptom onset date, admission date, existing underlying medical conditions, clinical suspicion of pneumonia (yes/no), chest radiograph findings (new abnormality, normal or not done), laboratory results (e.g., blood culture), and intensive care unit (ICU) admission. Due to infrequent ordering of bacterial cultures and recording of chest radiograph findings on case report forms, these variables were not included in the analysis. Nasopharyngeal swabs were collected from each SARI patient within 24 h of admission and tested for viral respiratory pathogens. Testing was performed using multiplex polymerase chain reaction (PCR) using the ResPlex II assay (v. 2.0) (Qiagen, Hilden, Germany) from 2010 to 2012 and the GenMark Respiratory Viral Panel assay from 2013 to 2014 (GenMark Diagnostics Inc., Carlsbad, CA, United States) [12, 13]. Viral targets included influenza viruses A and B, human metapneumovirus (HMPV), parainfluenza viruses (1–4), respiratory syncytial virus (RSV) A and B, rhinoviruses, coronaviruses (229E, OC43, NL63, HKU1) and adenoviruses.

We conducted a retrospective review of electronic medical records of each SARI case enrolled between week 40, 2010 and week 39, 2014 to obtain name, date of birth, discharge date and discharge status (dead/alive). On March 3, 2015, we queried the State of Arizona Office of Vital Records database to find matches between SARI patients and electronic death certificates using patient name, sex and date of birth. Variables obtained from death certificates and used in this analysis included date of death, underlying cause, contributing cause(s), place of death (inpatient, outpatient, hospice, decedents residence or other) and manner of death (natural or unnatural). Patients with death certificates listing a date of death beyond the date of hospital discharge were assumed to have died outside the sentinel hospital and were verified by place of death listed on the death certificate. Patients who died of unnatural causes were excluded from mortality calculations.

### Statistical analysis

Descriptive statistics of all SARI cases are reported as median values for continuous variables and frequencies and percentages for categorical variables. Mortality status was defined at 30-days post admission. The 30-day all-cause mortality from hospital admission date was calculated and reported as a percentage and 95% confidence interval. Deaths were categorized using *International Classification of Disease, Tenth Revision* (ICD-10) codes listed on death certificates for underlying and/or contributing cause of death. Cause of death was classified as pneumonia and influenza (ICD–10 codes J09–J18), respiratory and circulatory (ICD-10 codes J00–J99, I00–I99) or all-cause (all ICD-10 codes). Use of these categorizations for attributing cause of death due to influenza or other viral respiratory pathogens has been described elsewhere [14].

Predictors of mortality were identified by comparing patients who died with those who were alive at the 30-day post admission time point, using bivariate and multivariate logistic regression analysis. In hospitalized patients with pneumonia, a comparable condition to SARI, one study found that deaths within 30 days of admission were generally directly related to pneumonia, whereas deaths beyond 30 days were unrelated to pneumonia [15]. Additionally, the Centers for Medicare & Medicaid Services reported all-cause 30-day post admission mortality for patients with a principal diagnosis of pneumonia [16]. Therefore, we selected the 30-day post admission timeframe to capture SARI-related deaths while allowing our findings to be comparable with other research studies. Potential predictors examined in bivariate analysis included age, sex, race/ethnicity, length of hospital stay, time from symptom onset to hospital admission, ICU admission, presence of comorbidities (≥ 1, ≥ 2 and ≥ 3 comorbidities), individual comorbidities (hypertension, cardiac disease, lung disease, obesity, immunodeficiency and neurologic disorder), and the presence of individual viral pathogens (influenza viruses, parainfluenza viruses, coronaviruses, HMPV, RSV and rhinoviruses). Race/ethnicity was defined as a categorical variable with multiple levels. All other variables were defined as the presence or absence of the attribute. Independent predictors of mortality identified with a significance of *p* < 0.1 in bivariate analysis were included in the multivariate models. Non-significant factors in multivariate models were dropped using stepwise backward selection.

Kaplan-Meier curves for 30-day mortality were generated to display patient survival by independent predictors of mortality, and curves were compared using the log-rank test. Patients were censored at death or 30 days post hospital admission. Complete follow-up data were available for all patients at 30 days post hospital admission. All analyses were performed using SAS software version 9.3 and statistical significance was set at an alpha level of 0.05 (SAS Institute, Inc., Cary, NC).

## Results

A total of 306 SARI patients were admitted and enrolled by the two surveillance sites between week 40, 2010 and week 39, 2014; 48 did not meet the case definition and were excluded from further analyses leaving a total of 258 patients included in this analysis. Median age at admission was 63 years (range, 19–97 years) and 121 (47%) were women. One hundred thirty-one (51%) were white, non-Hispanic and 101 (39%) were Hispanic. The median time from symptom onset to hospital admission was four days (interquartile range (IQR); 2–6 days), the median duration of hospital stay was five days (IQR, 3–9 days); 96 (37%) patients were admitted to the ICU (Table 1).

**Table 1:** Characteristics of all SARI patients, and by status (alive/dead)

	All patients *n* = 258	Alive *n* = 228	Dead *n* = 30	*p* value
Demographic Characteristics	*n* (%)	*n* (%)	*n* (%)	
Age (median, range)	63 (19–97)	62 (19–97)	72 (35–88)	0.006
Age ≥ 65 years	135 (52%)	113 (50%)	22 (73%)	0.018
Sex (female)	121 (47%)	108 (47%)	13 (43%)	0.677
Race/Ethnicity				0.874
Asian	4 (2%)	3 (1%)	1 (3%)	
African American	6 (2%)	5 (3%)	1 (3%)	
Hispanic	101 (39%)	89 (40%)	12 (40%)	
Native American	16 (6%)	15 (7%)	1 (3%)	
White, non-Hispanic	131 (51%)	116 (49%)	15 (50%)	
Clinical Characteristics				
Duration of hospital stay (median; range)	5 (0–57)	5 (0–57)	6 (0–28)	0.482
Days to seek care (median; range)	4 (0–10)	4 (0–10)	3 (1–7)	0.288
Admission to intensive care unit	96 (37%)	74 (32%)	22 (73%)	<.0001
Clinical suspicion of pneumonia^a^	184 (71%)	159 (70%)	25 (83%)	0.175
≥ 3 Comorbidities	75 (29%)	64 (28%)	11 (36%)	0.332
≥ 1 Comorbidity	207 (80%)	180 (78%)	27 (90%)	0.165
Cardiac disease	69 (26%)	61 (26%)	8 (26%)	0.992
Hypertension	125 (48%)	108 (47%)	17 (56%)	0.340
Lung disease	84 (33%)	72 (31%)	12 (40%)	0.357
Obesity	29 (11%)	26 (11%)	3 (10%)	0.810
Immunodeficiency	23 (9%)	18 (8%)	5 (17%)	0.122
Neurological disorder	15 (6%)	13 (6%)	2 (6%)	0.832
Laboratory Results^b^	*n* = 253	*n* = 223	*n* = 30	
Influenza virus (A or B)	47 (19%)	43 (19%)	4 (13%)	0.464
Parainfluenza virus (1, 2 or 3)	13 (5%)	12 (5%)	1 (3%)	0.653
Coronavirus (OC43, HKU1, NL63, 229E)	14 (6%)	12 (5%)	2 (6%)	0.750
Human metapneumovirus	20 (8%)	18 (8%)	2 (6%)	0.754
Respiratory syncytial virus	8 (3%)	8 (3%)	n/a	
Rhinovirus	10 (4%)	9 (4%)	1 (3%)	0.870
Any viral pathogen^c^	113 (45%)	103 (46%)	10 (33%)	0.410

^a^Six SARI patients missing data on clinical suspicion of pneumonia; *n* = 252

^b^Five SARI patients did not have laboratory results; *n* = 253

^c^Three patients were co-infected; one with coronavirus HKU1/rhinovirus, one with coronavirus HKU1/human metapneumovirus and one with coronavirus 229E/rhinovirus

Overall, 207 (80%) patients had one or more underlying medical condition (27% had one, 23% had two, 18% had three and 11% had four or more). The most common underlying medical condition was hypertension (48%, *n* = 125) followed by chronic lung disease (33%, *n* = 84) and cardiovascular disease (26%, *n* = 69).

Nasopharyngeal specimens were collected and tested for 253 (98%) patients. Of the samples tested, 113 (45%) were positive for a viral respiratory pathogen. Nineteen percent of all the SARI samples and 42% of the positive samples (*n* = 47) had an influenza virus identified, 5% of the SARI and 12% of the positives (*n* = 13) had a parainfluenza virus, 8% of the SARI and 18% of the positives (*n* = 20) had a HMPV, 6% of the SARI and 12% of the positives (*n* = 14) had a coronavirus, and 3% of the SARI and 7% of the positives (*n* = 8) had a RSV (Table 1).

### Mortality

For all-cause mortality, 30/258 (12%, 95% CI 8–15%) patients died within 30 days of hospital admission [23/30 died in-hospital (77%)]. Among patients ≥ 65 years, 22/135 (16%, 95% CI 11–24%) died within 30 days of hospital admission [15/22 died in-hospital (68%)]. Among those who died, the median age at admission was 72 years (range: 35–88 years), the median duration of hospital stay was 6 days (range: 0–28 days), and the median time from symptom onset to death was 12 days (range: 4–32 days). Twenty-two of thirty (73%, 95% CI 58–89%) patients that died were admitted to the ICU [20/22 died in-hospital (90%)] and 27/30 (90%) patients had at least one underlying disease. An influenza virus was detected among 4/30 (13%) patients who died. All-cause mortality by influenza season was similar, with 5/36 deaths (14%) in the 2010–2011 season, 3/23 (13%) in 2011–2012, 6/44 (14%) in 2012–2013 and 16/125 (13%) in 2013–2014.

Underlying and contributing cause of death codes were listed on death certificates for 28 (93%) patients who died. Fourteen (50%) were classified as underlying pneumonia and influenza deaths and 14 (50%) as underlying respiratory and circulatory. All deaths were of natural causes.

### Predictors of 30-day mortality

**Fig. 1:**
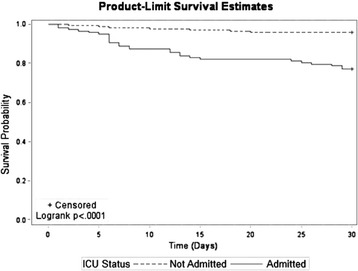
Kaplan Meier Estimate for 30-day survival post-hospital admission by age group

**Fig. 2:**
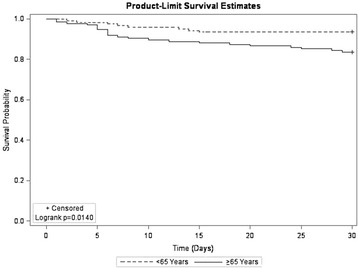
Kaplan Meier Estimate for 30-day survival post-hospital admission by ICU status

In bivariate analysis, only two factors were statistically significant in predicting 30-day mortality: admission to the ICU (OR 5.7; 95% CI 2.4–13.4) and age ≥ 65 years (OR 2.8; 95% CI 1.2–6.5) (Table 2). In multivariate analyses, ICU admission (OR 7.4; 95% CI 3.0–17.9) and age ≥ 65 years (OR 4.0; 95% CI 1.6–9.9) remained independent significant predictors of 30-day mortality. Kaplan-Meier curves of 30-day mortality by ICU admission and age group are shown in Figs. 1 and 2. ICU admission and age ≥ 65 years were associated with poorer survival at 30-days post admission (log-Rank test: p 0.01 and *p* < 0.0001, respectively).

**Table 2:** Bivariate and multivariate analyses for predictors of mortality

	Bivariate analysis		Multivariate analysis	
Factor	Odds Ratio (95% CI)	*p*-value	Odds Ratio (95% CI)	*p*-value
Age ≥ 65 years	2.8 (1.2–6.5)	0.01	4.0 (1.6–9.9)	0.002
Admission to intensive care unit	5.7 (2.4–13.4)	< 0.0001	7.4 (3.0–17.9)	< 0.0001
≥1 comorbidity	2.4 (0.7–8.2)	0.16	–	–
Hypertension	1.4 (0.6–3.1)	0.34	–	–
Chronic obstructive pulmonary disease	1.4 (0.7–3.1)	0.35	–	–
Duration of hospital stay	1.0 (0.9–1.1)	0.48	–	–
Antivirals given	0.6 (0.1–2.8)	0.54	–	–
Sex (female)	1.2 (0.5–2.5)	0.67	–	–
Race/Ethnicity^a^				
Asian	2.6 (0.3–26.4)	0.53	–	–
African American	0.5 (0.6–4.1)	0.42	–	–
Hispanic	1.0 (0.5–2.3)	0.91	–	–
Native American	1.5 (0.2–14.1)	0.69	–	–
Duration of hospital stay	1.0 (0.9–1.1)	0.48	–	–
Obese	0.8 (0.2–3.0)	0.81	–	–

^a^Referent group = White, non-Hispanic

## Discussion

In this Southern Arizona population, mortality among adult persons hospitalized with SARI was 9% and increased to 12% when a 30-day post-hospitalization window was considered. This demonstrated that the use of electronic vital records increased SARI-associated mortality estimates among adults by 30% by capturing deaths occurring outside the hospital; when limited to persons ≥ 65 years the increase was 47%. Age ≥ 65 years and admission to the ICU were significantly associated with mortality.

Given the lack of SARI studies in the United States, hospitalized adults with conditions such as community-acquired pneumonia (CAP) may be the best comparison group for SARI patients in this analysis. The 9% in-hospital mortality in this study was similar to one study of adults hospitalized with CAP in San Antonio, Texas (10%) but higher than in-hospital mortality in the Etiology of Pneumonia in the Community (EPIC) study (2%) [17, 18]. However, there are differences in the case definition between SARI and CAP that may affect direct comparisons. Additionally, in our surveillance, a larger proportion of SARI patients were admitted to the ICU (37% vs. 21%) and aged ≥ 65 years (56% vs. 36%) when compared to the proportions of CAP patients in the EPIC study, and this could have contributed to the higher observed mortality.

Globally, there are more SARI data for comparison. In-hospital mortality was 6% among adult SARI patients in eight African countries from 2009 to 2012 and 8% in a South African population with a high prevalence of HIV from 2012 to 2013 [19, 20]. In a study of SARI patients ≥ 18 years in Bogotá, Colombia, Remolina et al. reported higher in-hospital mortality of 15% [3]. One study of adult SARI patients in central China reported an overall mortality of 4% up to 30-days post discharge [21]. Again, the higher in-hospital mortality in our surveillance compared to other global SARI studies is not surprising given the 37% ICU admission and high median age of Arizona SARI patients.

The primary goal of this analysis was to better assess mortality in SARI patients by capturing additional deaths occurring soon after hospital discharge. Among adults ≥ 65 years hospitalized with CAP, previous studies have shown that nearly half of those who died did so after discharge [22]. Among Canadian adults hospitalized with CAP, Johnstone et al. reported a mortality of 12% at 30-days and 28% at one year follow-up [23]. A known limitation of the Arizona SARI surveillance program was that only in-hospital deaths were captured, and deaths occurring in patients discharged to hospices, nursing homes or private residences were not included. By using vital records, seven additional deaths occurring between hospital discharge and 30-days post admission were captured, supporting claims that mortality estimates for SARI were previously underestimated in Southern Arizona [11]. Although there is no gold standard for attributing cause of death to SARI, mortality studies that use vital records and administrative data to capture in-hospital and post discharge mortality may provide guidance for a more accurate way to estimate the proportion of deaths attributable to SARI. While hospital discharge practices vary globally, cross-country comparison of mortality studies may benefit from use of a 30-day post admission timeframe.

The independent risk factors for mortality that were identified in this analysis, age ≥ 65 years and ICU admission, are consistent with those found in a limited number of studies reporting risk factors for mortality in all SARI patients [20, 24]. Of 1790 adult and adolescent SARI patients in China, age ≥ 65 years was a significant risk factor for severe SARI outcomes (ICU admission or death) [21]. Among SARI patients aged ≥ 5 years in a South African population with high-HIV-prevalence, Cohen et al. found that ICU admission was a significant risk factor in bivariate analysis while increasing age was an independent risk factor in multivariate analysis [20]. As with previous studies assessing the effect of ICU admission on patient outcome, it is important to consider that our findings are likely subject to confounding by indication. This type of bias arises because patients with more severe illness are more likely to be admitted to the ICU, and more likely to die. Although we expected higher mortality given the 37% ICU admission in our analysis, one study of U.S. Medicare beneficiaries found lower 30-day mortality among patients with discretionary admission to the ICU after adjusting for distance to hospitals with high ICU admission rates for pneumonia [25].

Nineteen percent (47/253) of SARI patients tested positive for influenza viruses in this analysis. We can make some comparisons to other studies with the caveat that varying populations, years studied and enrollment methods may affect the findings. The proportion of influenza-positive SARI cases in our study was higher than reported in Kenya (10%) [26] and China (16%) [21], within range of eight African countries (5% in Tanzania to 26% in Madagascar) [19] and slightly lower than New Zealand (23%) [27]. In our surveillance system, the percent of SARI cases positive for an influenza virus varied by year from 5.6% to 20% and likely reflects the influenza activity of the dominant virus that year [11].

This analysis had several strengths. First, the inclusion of several seasons of surveillance data provided a more robust understanding of SARI and SARI-associated mortality in this region and showed consistent mortality across the seasons included in this analysis. Second, the geographic location of the surveillance program in the border region allowed for enrollment of a larger proportion of minority populations such as Hispanics and Native Americans, which may provide a better understanding of the burden of SARI in these populations. Lastly, this analysis benefited from the standardized data collection methods of SARI surveillance in conjunction with electronically available state death certificate data. The result was a surveillance database with a high level of detail on risk factors, clinical diagnoses and outcomes for each patient, but particularly for patients who died and had death certificate data.

This surveillance system has several limitations that may have affected the results of this analysis. First, the exclusion of patients from one sentinel hospital contributed to the relatively small sample size which may have limited the ability to detect potential risk factors of mortality, such as cardiovascular, renal, and neurological disease which have been shown by others to contribute to poor survival of patients with influenza and pneumonia in this population [28]. Second, exclusion of bacterial culture results and admitting chest x-ray findings prevented assessment of factors such as chest x-ray abnormalities and bacterial co-infections as risk factors for mortality. Third, the SARI patients included in this analysis may not be representative of the population of Southern Arizona where an estimated 22 million people crossed the northbound Arizona border in 2014 alone [11, 29]. Since sentinel sites in closer proximity to the U.S.-Mexico border were not included, the results of this analysis may not represent the burden of disease in the highly mobile border population. By limiting surveillance to large referral hospitals, we suspect there may be bias for certain risk groups, such as adults ≥ 65 years old, and more severe cases presenting later in the course of illness. The high frequency of ICU admission in our patients supports this hypothesis. Additionally, the sentinel sites used for this analysis are not major providers of pediatric care in Southern Arizona so a similar pediatric SARI mortality estimate remains unavailable in this population. Lastly, the linkage of death certificate data to SARI patients could have been more thorough had we searched death registries in nearby states and used unique identifiers such as social security number, had they been available.

## Conclusions

Surveillance for SARI in this region of the United States that has a highly fluid border population identified persons at high risk for severe respiratory illness and death, and these data may help target prevention strategies. The use of vital records provided additional information on the frequency and cause of mortality in patients with SARI and should be considered for use in other mortality studies when possible. Medical practitioners should be aware of a residual risk for mortality in SARI patients shortly after hospital discharge, especially in those aged ≥ 65 years or admitted to ICU, and should enhance post-discharge monitoring of these patients.

## Abbreviations

ADHS: Arizona Department of Health Services
BIDS: Border Infectious Disease Surveillance
CAP: Community-acquired pneumonia
CDC: Centers for Disease Control and Prevention
HMPV: Human metapneumovirus
ICD-10: International classification of disease, 10th revision
ICU: Intensive care unit
PCR: Polymerase chain reaction
RSV: Respiratory syncytial virus
SARI: Severe acute respiratory infection
WHO: World Health Organization
